# Association of the Healthy Dietary Index 2020 and its components with chronic respiratory disease among U.S. adults

**DOI:** 10.3389/fnut.2024.1402635

**Published:** 2024-07-03

**Authors:** Liu Zhiyi, Zhou Shuhan, Zhang Libing, Li Jiaqi, Ding Xin, Qin Lingxi, Shi Yuan-Mei, Zhang Hong, Nie Jiaqi, Li Hui, Fang Sanyou

**Affiliations:** ^1^School of Traditional Chinese Medicine, Hubei University of Chinese Medicine, Wuhan, China; ^2^Hospital of Stomatology Wuhan University, Wuhan, China; ^3^School of Pharmacy, Hubei University of Chinese Medicine, Wuhan, China; ^4^Taixing People’s Hospital, Taixing, China; ^5^Xiaogan Center for Disease Control and Prevention, Xiaogan, China

**Keywords:** HEI-2020, chronic bronchitis, emphysema, astham, NHANES

## Abstract

**Background:**

Chronic respiratory disease is an important public health problem in the United States and globally. Diet, an important part of a healthy lifestyle, is also relevant to chronic respiratory health. We aimed to explore the relationship between overall dietary quality and the risk of chronic respiratory disease (CRD), include chronic bronchitis (CB), emphysema and asthma.

**Method:**

A total of 4,499 United States adults were extracted from the National Health and Nutrition Examination Survey (NHANES) in 2017–2018. Diet quality was assessed using 2 day, 24 h dietary recall data and quantified as the Healthy Diet Index (HEI)-2020 score. Binary logistic regression models, restricted cubic splines (RCS) and generalized additive modeling (GAM), the weighted quartile sum (WQS) and qgcom models were used to assess the relationship between HEI-2020 scores and risk of CB, emphysema and asthma.

**Results:**

High HEI-2020 scores are associated with low risk of chronic respiratory disease (CB: 0.98, 0.97–0.99; emphysema: 0.98, 0.97–0.99; asthma: 0.98, 0.97–0.99) and consistent results across different dietary variable categorization (Tertile: CB: 0.58, 0.42–0.81; asthma: 0.51, 0.35–0.74; Quartile: CB: 0.57, 0.34–0.97; asthma: 0.56, 0.36–0.86) and different weighting models. Negative dose-response relationship between dietary quality and risk of chronic respiratory disease also shown in RCS and GAM models. The WQS and qgcom models also showed a healthy mixing effect of dietary components on respiratory disease, with high-quality proteins, vegetables, and fruits making the heaviest contributions.

**Conclusion:**

Higher HEI-2020 scores were associated with lower risk of CB, emphysema, and asthma. Following Dietary Guidelines for Americans 2020–2025 could support enhanced respiratory health.

## Introduction

Chronic bronchitis (CB) (1), emphysema (2) and asthma (3) are all among the most common chronic respiratory diseases (CRD) in the United States (4, 5). Asthma affects approximately 8% of U.S. adults (6). Chronic bronchitis and emphysema are both phenotypes of chronic obstructive pulmonary disease (COPD), which affects more than 15 million people across the U.S. and is the third leading cause of death in the U.S. and globally (6, 7).

Excluding genetic and allergic predispositions (8), the main risk factors for chronic respiratory diseases are infections or harmful substances in the environment (9). Prolonged inflammatory stress within the respiratory tract can induce tissue damage, culminating in the development of chronic pathologies (5). Regarding the prevention and treatment of chronic respiratory diseases, the relevant treatment guidelines have pointed out that there is currently no clear treatment drug, but more of a healthy lifestyle to improve the quality of life of patients (10, 11). The quality of diet is a pivotal component in the overall quality of life. In a study of dietary treatment of obese patients with COPD, rational restriction of energy intake was effective in improving patients’ BMI and muscle mass (12). This contributes to the development of guidelines for the health management of obese copd patients. The impact of diet on respiratory health may be derived from specific nutrients, specific foods, or a healthy diet, as also illustrated in the review on diet and COPD. Compared to nutrients, the protective effects of foods or healthy diets are more helpful in developing dietary guidelines (13).

In recent decades, there has been a growing body of research on diet and chronic disease (14–16), such as cognition, metabolic disease, cardiovascular disease. But few studies have explored the relationship between diet and respiratory and lung health. The healthy eating index (HEI) was developed and updated by the U.S. department of health and human services’ national cancer institute (NCI) and the U.S. department of agriculture (USDA) center for nutrition policy and promotion, as a quantitative measure of dietary quality for U.S. populations (17–19). It is a dietary quality indicator based on the Dietary Guidelines for Americans (DGA) (20). The HEI has been shown to relatively well reflect the dietary quality of the U.S. population and will be updated along with the DGA, which has gone through HEI-2005, HEI-2010, HEI-2015, and HEI-2020. HEI-2020 is currently the most recent version (21).

This study used the NHANES data to explore the relationship between HEI-2020 and the risk of CRD. Given the importance of diet to life and the high prevalence of respiratory diseases represented by asthma, chronic bronchitis, and emphysema in the U.S. population, this study has strong public health implications.

## Materials and methods

### Study sample

**Figure 1:**
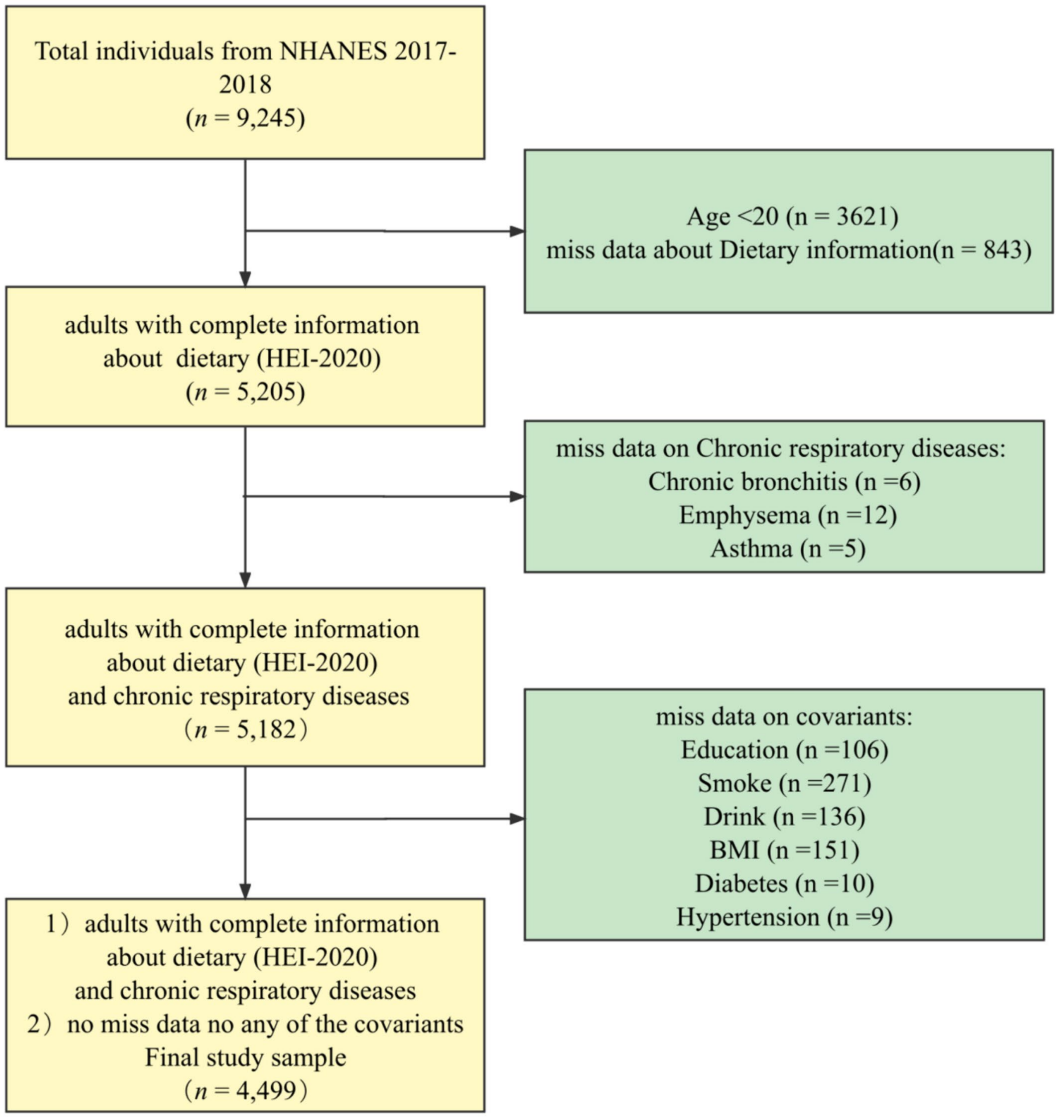
Flow chart.

The NHANES database is a regularly conducted cross-sectional study from the Centers for Disease Control and Prevention’s (CDC’s) National Center for Health Statistics (NCHS) that investigates nutritional intake and health-related conditions of populations in the U.S. (22). The NHANES utilizes a complex, multistage sampling design to make the survey results well-representative of populations across the U.S. The data for this study were obtained from NHANES 2017–2018. The final sample, *n* = 4,499, was weighted to represent 103.6 million non-institutionalized adult U.S. population, and the process can be seen in Figure 1.

#### Diet quality

The HEI-2020 is designed to evaluate adherence to the Dietary Guidelines for Americans (DGA) for the years 2020–2025, encompassing 13 distinct components: adequacy components (total vegetables, greens and beans, total fruits, whole fruits, whole grains, dairy, total protein foods, seafood, and plant proteins, as well as fatty acids) and moderation components (sodium, refined grains, saturated fats, and added sugars) (21). Each component has been assigned unique weights and distinct maximum scores. The HEI-2020 scores are scaled from 0 to 100, where a higher score indicates superior diet quality. For weighted Scott–Rao chi-square tests and weighted logistic regressions, HEI-2020 was used as continuous variable and categorical variable. Quartiles were used to categorize the HEI-2020 score into four groups, and record them as Q1(reference group), Q2, Q3, Q4 (23). Tertiles were also used to categorize the HEI-2020 score into three groups, and record them as T1(reference group), T2,T3 (24).

### Chronic respiratory diseases

Three chronic respiratory diseases (chronic bronchitis (CB), emphysema, and asthma) were selected as outcome variables in this study. CB, emphysema, and asthma were all defined as non-patient groups and patient groups (25).

### Covariates

In order to reduce the influence of confounding factors and obtain more reliable results, we referred to the relevant literature and selected demographic-sociological covariates [Sex, Age (26), Race (27), Education (28), Family income (29)], behavioral covariates [BMI status (30), Physical activity (31), Smoking status (32), Drinking status (15)], and chronic disease covariates [Hypertension (33), Diabetes (34)]. Physical activity is computationally defined through metabolic equivalent (MET). MET is the oxygen consumption required to maintain resting metabolism (35). According to the Physical Activity Questionnaire (PAQ) survey in NHANES included vigorous work-related activity (8MET), moderate work-related activity (4MET), walking or bicycling for transportation (4MET), vigorous amateur physical activity (8MET), and moderate amateur physical activity (4MET). Calculation formula (36):


$$\begin{array}{l} \mathrm{PA}\;\left(\mathrm{MET}-h/\mathrm{wk}\right)=\mathrm{MET}\times \mathrm{weekly frequency}\\ \;\;\;\;\;\;\;\;\;\;\;\;\;\;\;\;\;\;\;\;\;\;\;\;\;\;\;\;\;\;\;\;\;\;\;\;\;\;\;\;\;\;\;\;\;\;\;\;\times \mathrm{physical activity duration}. \end{array}$$

### Statistical analysis

The characteristics of different groups were tested using the *χ*^2^ tests and *t*-test. Weighted steps forward (likelihood ratio) binary logistic regression models were employed to evaluate the association between HEI-2020 scores and the risk of chronic bronchitis, emphysema, and asthma. Restricted cubic splines (RCS) were utilized to examine the dose-response relationship between HEI-2020 scores and chronic respiratory diseases conditions. The second approach is generalized additive modeling (GAM) regression, which assumes a smooth and possibly nonlinear association between HEI-2020 scores and chronic respiratory disease (37, 38). The effects of combined exposure to 13 dietary components of the HEI-2020 were assessed using the weighted quartile sum (WQS) regression model (39). The quantile G-computation method (qgcomp) was used to explore the joint and independent effects of HEI-2020 scores on the risk of chronic respiratory disease (40, 41). Examine the reliability of the results between dietary quality and CRD risk, with components of dietary quality included in the WQS and qgcom models as individual component scores rather than total scores. To analyze the overall health mixing effect of dietary components on CRD risk and the proportion of health contribution of each dietary component in the organized dietary structure.

Sensitivity analyses were applied to this study; the study sample was used as an unweighted sample, a directly weighted sample, and a weighted sample corrected for the three study populations by the Taylor Linear Method; HEI-2020 scores were applied as a continuous variable, a quartile categorized variable, and a tertile categorized variable included in a generalized linear model. RCS were used to test the dose-response relationship between HEI when used as a continuous variable and risk of chronic respiratory disease. GAM was used as a sensitivity analysis to validate the RCS results. The WQS model was used in order to further validate the HEI-2020 score with respect to chronic respiratory disease risk and to explore the weighting of dietary components contributing to respiratory health. Qgcomp modeling was used as a sensitivity analysis to validate the results of the WQS model.

All statistical tests were two-sided, and significance was considered at *p* < 0.05. All statistical analysis were performed with the R (version 4.1.2). RCS was implemented with the R package “rms” (version 6.3-0). GAM was implemented with the R package “gam” (version 1.22-2). WQS was implemented with the R package “gWQS” (version 3.0.4). qgcom was implemented with the R package “qgcom” (version 2.15.2).

## Results

### Characteristics of the study population

After screening the sample, a total sample of *n* = 4,499 was included. Weighted transformations represent 103.6 million of the U.S. non-institutionalized adult population of which 48.8% were male and 51.2% were female, with a mean HEI-2020 score of 52.92 ± 13.99 (Table 1). As seen in the characterization between the healthy population and the CRD population, adults with chronic respiratory disease were more likely to be older, have lower incomes, obesity, less physically active, have chronic illnesses, lower dietary quality.

**Table 1:** The population characteristics among U.S. adults by the three chronic respiratory diseases.

Characteristics (Weighted%)	Total	Chronic bronchitis		*p*-value	Emphysema		*p*-value	Asthma		*p*-value
		Non-patients	Patients		Non-patients	Patients		Non-patients	Patients	
*n*	4,499	4,185	314		4,424	75		3,808	691	
Sex				0.09			0.782			0.013
Male	2,206 (48.8%)	2073 (49.1%)	133 (40.3%)		2,160 (48.5%)	46 (46.2%)		1909 (49.8%)	297 (40.9%)	
Female	2,293 (51.2%)	2,112 (50.9%)	181 (59.7%)		2,264 (51.5%)	29 (53.8%)		1899 (50.2%)	394 (59.1%)	
Age group				<0.001			<0.001			0.16
20–39 y	1,359 (35.2%)	1,307 (36.4%)	52 (19.3%)		1,358 (35.3%)	1 (1.1%)		1,121 (34.4%)	238 (39.9%)	
40–59 y	1,445 (36.1%)	1,330 (36%)	115 (38%)		1,424 (36.3%)	21 (32%)		1,222 (36.2%)	223 (35.8%)	
60–79 y	1,427 (25.2%)	1,309 (24.3%)	118 (37.1%)		1,382 (25.2%)	45 (61.1%)		1,220 (25.7%)	207 (22.3%)	
80+ y	268 (3.5%)	239 (3.3%)	29 (5.6%)		260 (3.3%)	8 (5.8%)		245 (3.8%)	23 (2%)	
Race				0.088			0.16			0.108
Hispanic	997 (14.9%)	954 (15.3%)	43 (9%)		990 (14.1%)	7 (4.8%)		862 (15.1%)	135 (13.5%)	
Non-Hispanic White	1,628 (64.2%)	1,465 (63.8%)	163 (69.6%)		1,584 (64.6%)	44 (72.3%)		1,381 (64.8%)	247 (61.2%)	
Non-Hispanic Black	1,064 (11.1%)	996 (11.2%)	68 (10.1%)		1,051 (11.1%)	13 (5.8%)		859 (10.4%)	205 (15.2%)	
Other Race	810 (9.8%)	770 (9.7%)	40 (11.3%)		799 (9.9%)	11 (17.2%)		706 (9.8%)	104 (10.1%)	
Education				0.178			0.009			0.061
<High School	815 (10%)	759 (9.9%)	56 (11.3%)		799 (11%)	16 (10.8%)		702 (10.1%)	113 (9.8%)	
High school/GED	1,096 (27.8%)	1,004 (27.3%)	92 (33.7%)		1,062 (27.2%)	34 (57%)		913 (28%)	183 (26.4%)	
College/AA degree	1,489 (30.8%)	1,366 (30.6%)	123 (33.4%)		1,469 (30.3%)	20 (16%)		1,227 (29.5%)	262 (37.7%)	
College or above	1,099 (31.5%)	1,056 (32.2%)	43 (21.7%)		1,094 (31.3%)	5 (16.3%)		966 (32.4%)	133 (26.1%)	
Family income				0.002			0.182			0.055
0–130 FPL	1,102 (17.4%)	995 (16.8%)	107 (26%)		1,075 (17.1%)	27 (25.7%)		891 (16.5%)	211 (22.7%)	
>130–350 FPL	1,648 (32.6%)	1,517 (32%)	131 (41.5%)		1,614 (32.3%)	34 (43.4%)		1,406 (32.4%)	242 (34%)	
>350 FPL	1749 (50%)	1,673 (51.3%)	76 (32.5%)		1735 (55%)	14 (30.9%)		1,511 (51.2%)	238 (43.3%)	
BMI				<0.001			0.19			0.004
Normal weight	1,084 (25%)	1,032 (25.9%)	52 (12.8%)		1,059 (22%)	25 (26.8%)		949 (25.5%)	135 (22.2%)	
Underweight	67 (1.6%)	60 (1.4%)	7 (4.1%)		63 (1.1%)	4 (7.4%)		54 (1.3%)	13 (3%)	
Overweight	1,426 (30.1%)	1,341 (30.1%)	85 (30.3%)		1,410 (30.3%)	16 (29.1%)		1,251 (31.4%)	175 (22.5%)	
Obese	1922 (43.3%)	1752 (42.7%)	170 (52.8%)		1892 (43.4%)	30 (36.7%)		1,554 (41.8%)	368 (52.2%)	
Drink status				0.002			0.113			0.345
No	447 (6.9%)	429 (7.2%)	18 (3.3%)		444 (6.1%)	3 (2.6%)		389 (7.1%)	58 (6%)	
Yes	4,052 (93.1%)	3,756 (92.8%)	296 (96.7%)		3,980 (93.9%)	72 (97.4%)		3,419 (92.9%)	633 (94%)	
Smoke status				<0.001			<0.001			0.477
Never smoker	2,578 (57.5%)	2,472 (59.4%)	106 (30.1%)		2,571 (57.5%)	7 (4.8%)		2,219 (57.9%)	359 (54.9%)	
Former smoker	1,096 (25.4%)	994 (24.6%)	102 (36.2%)		1,062 (25.2%)	34 (54.2%)		904 (24.9%)	192 (28.2%)	
Current smoker	825 (17.1%)	719 (15.9%)	106 (33.7%)		791 (17.1%)	34 (41%)		685 (17.2%)	140 (17%)	
PA				0.007			0.001			0.2
No	1,139 (20.7%)	1,034 (20.1%)	105 (28.4%)		1,103 (20.2%)	36 (47.2%)		958 (20%)	181 (24.4%)	
Low	1735 (40%)	1,617 (39.8%)	118 (42.4%)		1717 (44%)	18 (32.7%)		1,473 (39.9%)	262 (40.1%)	
High	1,625 (39.4%)	1,534 (40.1%)	91 (29.2%)		1,604 (39.3%)	21 (20.1%)		1,377 (40%)	248 (35.5%)	
Diabetes				<0.001			0.108			0.223
No	3,663 (86.2%)	3,442 (87.1%)	221 (73%)		3,610 (86.9%)	53 (73.4%)		3,121 (86.5%)	542 (84.1%)	
Yes	836 (13.8%)	743 (12.9%)	93 (27%)		814 (13.1%)	22 (26.6%)		687 (13.5%)	149 (15.9%)	
Hypertension				0.002			0.034			0.005
No	2,788 (67.6%)	2,639 (68.8%)	149 (51.5%)		2,754 (67.7%)	34 (43.1%)		2,407 (68.8%)	381 (60.9%)	
Yes	1711 (32.4%)	1,546 (31.2%)	165 (48.5%)		1,670 (32.3%)	41 (56.9%)		1,401 (31.2%)	310 (39.1%)	
HEI continuous (SD)	52.92 (13.99)	53.19 (14.00)	49.39 (13.45)	0.008	53.03 (14.00)	46.60 (12.14)	0.485	53.28 (14.08)	50.94 (13.30)	0.002
HEI Category with Tertile				0.007			0.311			<0.001
T_1_	1,498 (34.6%)	1,366 (34.1%)	132 (41.9%)		1,460 (34.3%)	38 (36.9%)		1,228 (32.9%)	270 (44.3%)	
T_2_	1,498 (33.6%)	1,393 (33.3%)	105 (37.2%)		1,470 (33.3%)	28 (44.5%)		1,260 (33.4%)	238 (34.7%)	
T_3_	1,503 (31.8%)	1,426 (32.6%)	77 (20.9%)		1,494 (31.3%)	9 (18.6%)		1,320 (33.7%)	183 (21%)	
HEI category with quartile				0.063			0.478			0.003
Q_1_	1,125 (26.6%)	1,022 (26%)	103 (35.2%)		1,097 (26.2%)	28 (28.1%)		928 (25.6%)	197 (32.9%)	
Q_2_	1,124 (24.8%)	1,041 (24.7%)	83 (26.3%)		1,101 (24.2%)	23 (31.4%)		925 (24.1%)	199 (29.2%)	
Q_3_	1,125 (25.1%)	1,051 (25.3%)	74 (22.7%)		1,107 (25.2%)	18 (28.4%)		972 (25.6%)	153 (22.2%)	
Q_4_	1,125 (23.4%)	1,071 (24%)	54 (15.7%)		1,119 (23.2%)	6 (12%)		983 (24.8%)	142 (15.7%)	

FPL, family income to poverty; BMI, body mass index; PA, physical activity; *p*, *p* value.

### Higher HEI-2020 scores is associated with a lower risk of CRD

A multifactorial stepwise logistic regression model was employed to examine the association between HEI-2020 scores and risk of CRD. All models adjusted for demographic-sociological covariates (Sex, Age, Education, Family income), behavioral covariates (BMI status, Physical activity, Smoking status, Drinking status), and chronic disease covariates (Hypertension, Diabetes). Table 2 shown relationship between higher HEI-2020 scores and a lower risk of CRD in both the three kinds of binary logistic regression model. Whether the study sample was weighted or not, the HEI-2020 score showed a healthy effect on the risk of all three CRD when used as a continuous variable (CB: 0.98, 0.97–0.99; emphysema: 0.98, 0.97–0.99; asthma: 0.98, 0.97–0.99). This relationship was not significantly altered after tertile and quartile categorization of HEI-2020 scores (Tertile: CB: 0.58, 0.42–0.81; asthma: 0.51, 0.35–0.74; Quartile: CB: 0.57, 0.34–0.97; asthma: 0.56, 0.36–0.86). It suggests that higher HEI-2020 scores are correlated with low risk of CRD. Suggests that neither changes in the proportion of the population in the study sample nor different ways of defining HEI-2020 (dietary quality) affect the association of high HEI-2020 scores with low CRD.

**Table 2:** Relationship between HEI-2020 and chronic respiratory disease among adults aged 20 years or older.

Variable	OR (95%CI)		
	Unweighted	Crude weighted	Adjusted weighted
Chronic bronchitis			
HEI continuous	0.98 (0.97,0.99)*	0.98 (0.96,0.99)*	0.98 (0.97,0.99)*
HEI Category (ref T1)			
T2	0.87 (0.66,1.15)	0.91 (0.65,1.27)	0.97 (0.7,1.35)
T3	0.69 (0.51,0.95)*	0.52 (0.35,0.78)*	0.58 (0.42,0.81)*
HEI Category (ref Q1)			
Q2	0.86 (0.63,1.18)	0.79 (0.55,1.13)	0.83 (0.57,1.2)
Q3	0.81 (0.59,1.13)	0.67 (0.4,1.1)	0.71 (0.43,1.16)
Q4	0.65 (0.45,0.93)*	0.49 (0.26,0.89)*	0.57 (0.34,0.97)*
Emphysema			
HEI continuous	0.97 (0.95,0.99)*	0.98 (0.96,0.99)*	0.98 (0.97,0.99)*
HEI Category (ref T1)			
T2	0.79 (0.46,1.33)	1.25 (0.83,1.87)	1.36 (0.86,2.16)
T3	0.29 (0.14,0.61)*	0.54 (0.14,2.05)	0.64 (0.19,2.22)
HEI Category (ref Q1)			
Q2	0.82 (0.45,1.47)	1.2 (0.72,2.01)	1.15 (0.62,2.14)
Q3	0.76 (0.40,1.43)	1.07 (0.57,2.03)	1.18 (0.65,2.16)
Q4	0.27 (0.10,0.65)	0.48 (0.09,2.5)	0.6 (0.12,3.07)
Asthma			
HEI continuous	0.99 (0.98,0.99)*	0.98 (0.97,0.99)*	0.98 (0.97,0.99)*
HEI Category (ref T1)			
T2	0.90 (0.74,1.10)	0.77 (0.58,1.03)	0.79 (0.59,1.04)
T3	0.74 (0.59,0.92)*	0.46 (0.33,0.66)*	0.51 (0.35,0.74)*
HEI Category (ref Q1)			
Q2	1.08 (0.86,1.35)	0.94 (0.66,1.34)	0.96 (0.69,1.35)
Q3	0.83 (0.65,1.052)	0.67 (0.48,0.94)	0.73 (0.51,1.04)
Q4	0.83 (0.64,1.07)	0.49 (0.32,0.75)*	0.56 (0.36,0.86)*

*p < 0.05, T, Tertile; Q, Quartile. Crude weighed = add weight to unweighted. Adjusted weighted = Taylor line correction for Crude weighted. All models adjusted for sex, age, education, family income, BMI status, physical activity, smoking status, drinking status, hypertension, diabetes.

### Dose-response relationship between HEI-2020 scores and risk of CRD

**Figure 2:**
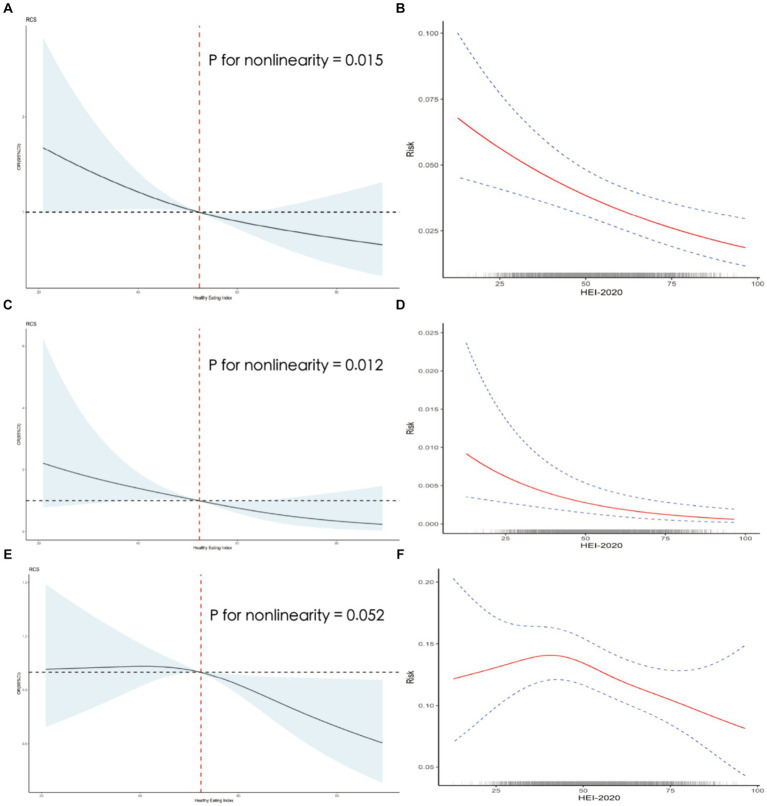
Dose-response association between HEI-2020 (in continues) and chronic bronchitis/emphysema/asthma using restricted cubic splines (RCS) and generalized additive modeling (GAM) regression. The chronic bronchitis models of RCS **(A)** and GAM **(B)**. The emphysema models of RCS **(C)** and GAM **(D)**. The asthma models of RCS **(E)** and GAM **(F)**. All models adjusted for sex, age, education, family income, BMI status, physical activity, smoking status, drinking status, hypertension, diabetes.

RCS was used to explore the dose–response relationship between HEI-2020 scores and the risk of three CRD. GAM was used as a sensitivity analysis to validate the results. The RCS results showed a negative dose-relative relationship between HEI and the risk of the three CRD in Figure 2; meanwhile, the GAM results also showed a same tendency (Figure 2).

### Mixed effects of 13 dietary components on CRD

Table 3 shows the mixed effects of the 13 dietary components of the HEI-2020 on the risk of CRD in the model of WQS and the model of qgcomp. The results of the WQS and qgcomp models showed that HEI-2020 scores still showed a significant protective effect between the three CRD when they were used as a mixing variable. Suggesting that the correlation between high dietary quality and low chronic respiratory risk is not altered by the inclusion of model definitions. The 13 dietary components of HEI-2020 have a healthy mixing effect on the respiratory tract. And the size of the contribution varied among the different food components.

**Table 3:** Relationship between the mixed effects of the 13 dietary components of the HEI-2020 and chronic respiratory disease among adults aged 20 years or older.

Disease outcome	OR (95%CI)	*p*-value
WQS		
Chronic bronchitis	0.92 (0.86,0.098)	0.04
Emphysema	0.84 (0.71,0.98)	0.04
Asthma	0.95 (0.92,0.099)	0.01
qgcomp		
Chronic bronchitis	0.49 (0.33,0.074)	0.00
Emphysema	0.39 (0.01,1.55)	0.18
Asthma	0.37 (0.22,0.66)	0.00

All models adjusted for sex, age, education, family income, BMI status, physical activity, smoking status, drinking status, hypertension, diabetes.

**Figure 3:**
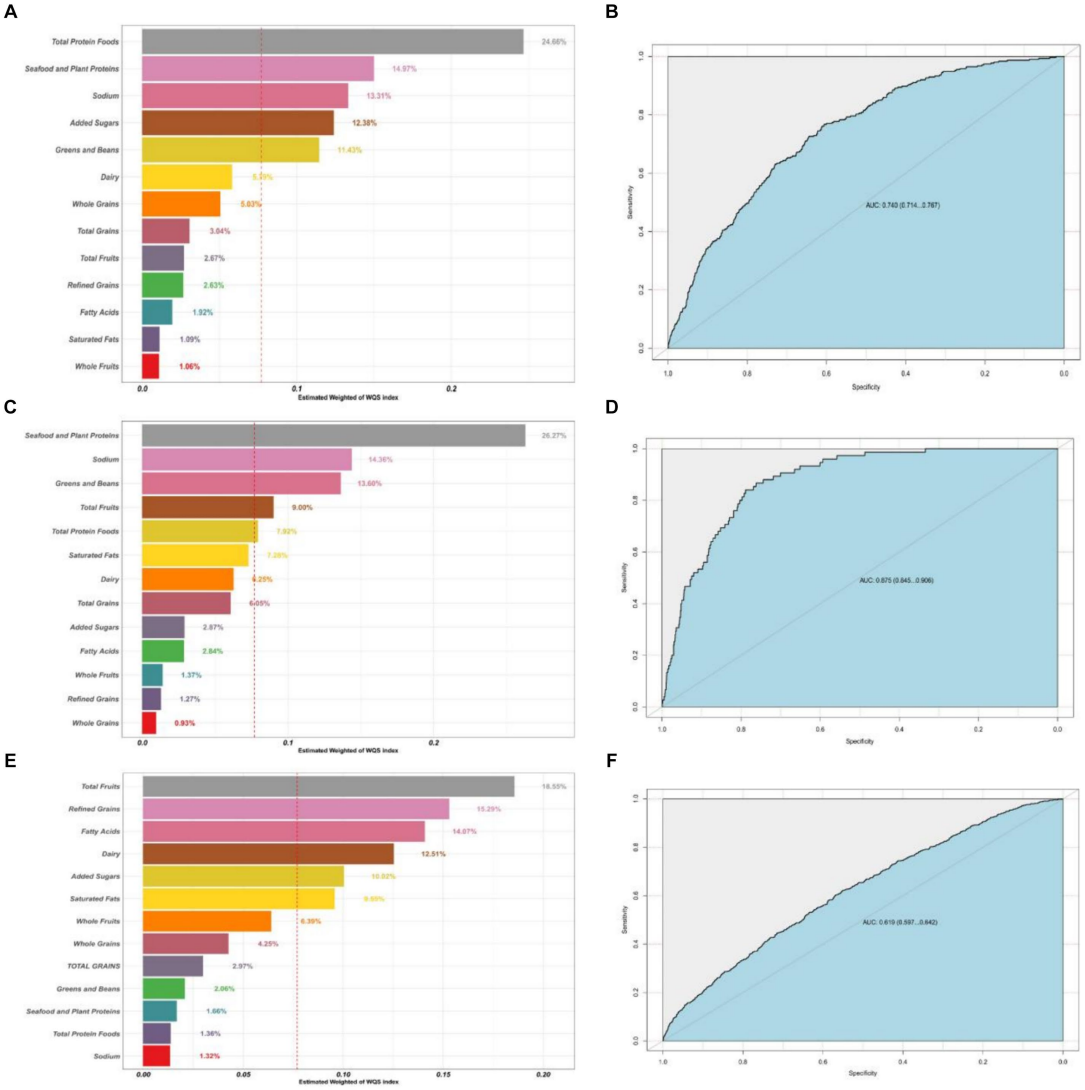
Contribution weights of dietary components in the WQS model of CB **(A)** and the AUCs of the WQS models **(B)**. Contribution weights of dietary components in the WQS model of emphysema **(C)** and the AUCs of the WQS models **(D)**. Contribution weights of dietary components in the WQS model of asthma **(E)** and the AUCs of the WQS models **(F)**. All models adjusted for sex, age, education, family income, BMI status, physical activity, smoking status, drinking status, hypertension, diabetes.

Figure 3 shows the results of the mixed effects of dietary components on the risk of CRD in the WQS model. WQS results showed that Total Protein Foods, Seafood and Plant Proteins and Sodium had the largest health contribution group in the CB model, 24.66, 14.97, 13.31%, respectively; Seafood and Plant Proteins and Sodium had the largest health contribution group in the emphysema model, 26.27, 14.36%, respectively; and in the asthma model, Total Fruits, Refined Grains, and Fatty Acids made the greatest health contributions 18.55, 15.29, and 14.07%.

**Figure 4:**
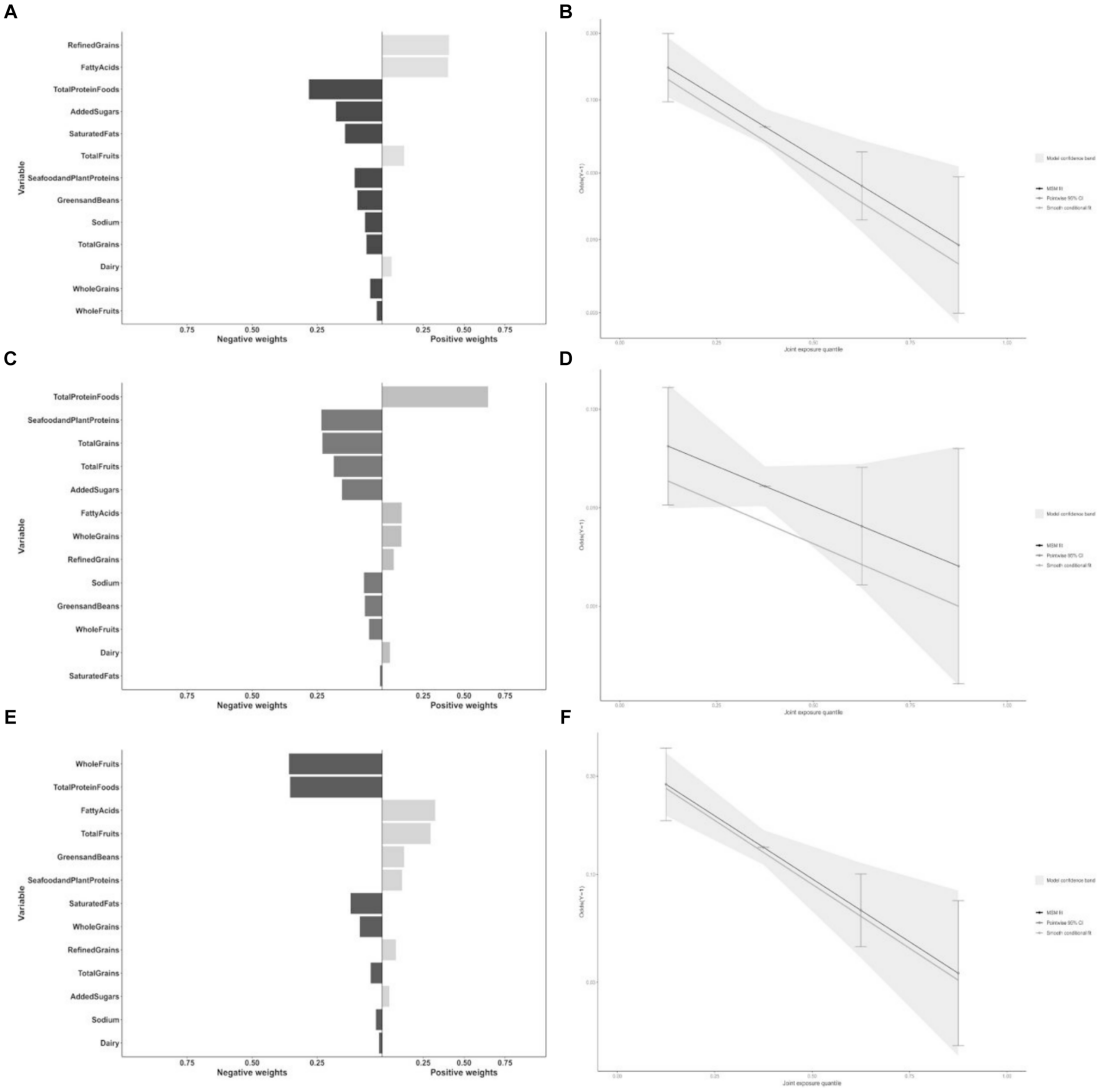
Contribution weights of dietary components in the qgcomp model of CB **(A)** and the trends visualization of qgcomp model **(B)**. Contribution weights of dietary components in the qgcomp model of emphysema **(C)** and the Trends Visualization of qgcomp model **(D)**. Contribution weights of dietary components in the qgcomp model of asthma **(E)** and the Trends Visualization of qgcomp model **(F)**. All models adjusted for sex, age, education, family income, BMI status, physical activity, smoking status, drinking status, hypertension, diabetes.

Figure 4 shows the results of the mixed effects of dietary components on the risk of CRD in the qgcomp model. The results showed that the dietary components whose health contribution was the greatest in the three models of CRD were, Total Protein Foods; Seafood and Plant Proteins; and Whole Fruits, respectively.

## Discussion

Our study included 4,499 U.S. adult subjects who could represent 103.6 million non-institutionalized adult U.S. population after appropriate weighting. We have found, after multiple model validations, that higher dietary quality, as represented by the HEI-2020 score, is associated with lower risk of the three CRD (CB, emphysema and asthma) in the U.S. adult population. Among different population proportions, different HEI classification methods, and different analytical models, this result is stable. Negative dose-response relationships between HEI-2020 scores and risk of the three CRD were found in both the RCS and GAM models. The WQS and qgcomp similarly found a health effect of the HEI-2020 mixed effect for the three CRD. It suggesting that the relationship between dietary quality and risk of CRD is similarly independent of HEI-2020 in terms of model type and inclusion criteria in the model. In the mixed health effects of 13 dietary components on respiratory health from HEI-2020, Total Protein Foods were found to make the greatest health contribution to CB risk, Seafood and Plant Proteins made the greatest health contribution to emphysema risk; and fruits made the greatest health contribution to asthma risk.

Western dietary patterns rich in preserved and processed meat foods have also been associated with a higher risk of chronic respiratory disease, possibly due to the higher inflammatory stress associated with high sugar and salt intake associated with nitrite in processed foods (42, 43). A study of diets and COPD risk in U.S. adults found that the better the quality of the diet represented by the Dietary Approaches to Stop Hypertension (DASH) score and Mediterranean diets score, the lower the risk of COPD (44). Similarly, the health contributions of DASH diets and Mediterranean diets to lung health have likewise been reported in other studies (45–48). A study by Alwarith et al. also found that a plant-based diet prevented asthma attacks and improved symptoms, possibly because fruit and vegetable intake may mediate the release of cytokines, free radical damage, and immune responses associated with the onset and development of asthma (49). These results are consistent with the results of the present study. In contrast to these dietary patterns, the dietary patterns represented by the HEI, established by official U.S. agencies, are more appropriate for the U.S. population. The HEI’s quantification of dietary intake in terms of energy density is also more suitable for individual use.

In the present study, the dose–response relationship between dietary score and disease risk was also examined and sensitivity analyzed, and the RCS and GAM results showed a negative dose-response between dietary score and respiratory disease risk. This is also in line with the general perception of healthy diets in good health. Similarly, we used the mixed effects of the 13 dietary components to replace the total HEI-2020 score to further validate the association between dietary quality and respiratory disease risk, while exploring the contribution of different food groups in it. The results of WQS and G-computation modeling showed that there was a healthy mixing effect of the 13 dietary components of the HEI-2020 in respiratory disease risk with the trend of the relationship between HEI-2020 scores and respiratory disease risk. Appropriately elevated intake of total protein foods, seafood and plant proteins, and fruits reduced disease risk of CRD.

An observational study in Iceland found that low protein intake was associated with risk of malnutrition, length of hospitalization, and mortality in COPD patients (50). Another observational study from the National Lung Hospital (NLH) in Hanoi, Vietnam, also found a high prevalence of malnutrition among COPD patients, and that increasing energy- and protein-rich foods may help to improve the nutritional status and quality of life of Vietnamese COPD patients (51). Nutritional therapy has been shown to be effective in maintaining and improving muscle strength and exercise tolerance in malnourished COPD patients (52). Low body weight and low fat-free body weight (FFM) have been identified as poor prognostic factors in patients with COPD, and relevant populations and clinical trials have found that high-quality protein or supplementation with essential amino acids can increase FFM index and improve arterial oxygen levels (53, 54). A study of protein absorption and utilization in patients with COPD identified low protein intake, systemic inflammation, and hypertension as risk factors for lower postabsorptive protein balance in patients with COPD, and lower post-absorptive protein balance was associated with markers of poorer daily physical function (55). Two large cohort studies in the United States found higher marine fish intake was associated with a lower risk of COPD (56). These studies all reflect the healthy role of protein, seafood and fruit and vegetable intake in respiratory health.

This study has certain strengths, most notably, it utilized a multi-model sensitivity analysis design, with different sample population proportions, different ways of defining variables, and different models validating each other to make the results more realistic and reliable. The second is the use of a large U.S. Nutrition and Health Survey database, which is reliable and representative; and lastly, the use of the latest version of the Healthy Dietary Index, HEI-2020, which is based on DGA2020–2025, which is representative of the U.S. population’s dietary intake. As well, there are shortcomings in this study, the largest being that this was a cross-sectional study and could not validate the causal link between dietary quality and the risk of chronic respiratory disease. Secondly, definition of disease from my self-report, with possible recall bias that cannot be eliminated. Third, the study focused on the U.S. adult population, with insufficient extrapolation to pre-adult U.S. and non-U.S. populations.

The study found that high HEI-2020 scores were associated with low chronic respiratory risk, suggesting that improving the quality of the diet itself by following DGA 2020–2025 could help prevent the occurrence and exacerbation of chronic respiratory diseases.

## Conclusion

Higher HEI scores are associated with lower risk of chronic respiratory disease, and this trend is more stable and reliable. More attention should be given to overall dietary intake of high-quality protein, seafood, vegetables, and fruits.

## Data availability statement

The original contributions presented in the study are included in the article/supplementary material, further inquiries can be directed to the corresponding author.

## Author contributions

LZ: Funding acquisition, Writing – original draft, Writing – review & editing. ZS: Data curation, Writing – original draft. ZL: Software, Writing – original draft. LJ: Data curation, Supervision, Writing – original draft. DX: Investigation, Software, Writing – original draft. QL: Investigation, Software, Writing – original draft. SY-M: Data curation, Visualization, Writing – review & editing. ZH: Data curation, Supervision, Writing – review & editing. NJ: Formal analysis, Methodology, Software, Supervision, Writing – review & editing. LH: Writing – original draft, Writing – review & editing. FS: Writing – review & editing.
